# Digital Transformation in Smart Farm and Forest Operations Needs Human-Centered AI: Challenges and Future Directions

**DOI:** 10.3390/s22083043

**Published:** 2022-04-15

**Authors:** Andreas Holzinger, Anna Saranti, Alessa Angerschmid, Carl Orge Retzlaff, Andreas Gronauer, Vladimir Pejakovic, Francisco Medel-Jimenez, Theresa Krexner, Christoph Gollob, Karl Stampfer

**Affiliations:** 1 Human-Centered AI Lab, Institute of Forest Engineering, Department of Forest and Soil Sciences, University of Natural Resources and Life Sciences Vienna, 1190 Wien, Austria; anna.saranti@human-centered.ai (A.S.); alessa.angerschmid@human-centered.ai (A.A.); carlorge.retzlaff@dai-labor.de (C.O.R.); 2 xAI Lab, Alberta Machine Intelligence Institute, University of Alberta, Edmonton, AB T5J 3B1, Canada; 3 DAI Lab, Technical University Berlin, 10623 Berlin, Germany; 4 Institute of Agricultural Engineering, Department of Sustainable Agricultural Systems, University of Natural Resources and Life Sciences Vienna, 1180 Wien, Austria; andreas.gronauer@boku.ac.at (A.G.); vladimir.pejakovic@boku.ac.at (V.P.); francisco.medel-jimenez@boku.ac.at (F.M.-J.); theresa.krexner@boku.ac.at (T.K.); 5 Institute of Forest Growth, Department of Forest and Soil Sciences, University of Natural Resources and Life Sciences Vienna, 1180 Wien, Austria; christoph.gollob@boku.ac.at; 6 Institute of Forest Engineering, Department of Forest and Soil Sciences, University of Natural Resources and Life Sciences Vienna, 1180 Wien, Austria; karl.stampfer@boku.ac.at

**Keywords:** sensors, cyber-physical systems, machine learning, artificial intelligence, human-centered AI, smart farming, smart forestry, precision farming, precision forestry, AI for good

## Abstract

The main impetus for the global efforts toward the current digital transformation in almost all areas of our daily lives is due to the great successes of artificial intelligence (AI), and in particular, the workhorse of AI, statistical machine learning (ML). The intelligent analysis, modeling, and management of agricultural and forest ecosystems, and of the use and protection of soils, already play important roles in securing our planet for future generations and will become irreplaceable in the future. Technical solutions must encompass the entire agricultural and forestry value chain. The process of digital transformation is supported by cyber-physical systems enabled by advances in ML, the availability of big data and increasing computing power. For certain tasks, algorithms today achieve performances that exceed human levels. The challenge is to use multimodal information fusion, i.e., to integrate data from different sources (sensor data, images, *omics), and explain to an expert why a certain result was achieved. However, ML models often react to even small changes, and disturbances can have dramatic effects on their results. Therefore, the use of AI in areas that matter to human life (agriculture, forestry, climate, health, etc.) has led to an increased need for trustworthy AI with two main components: explainability and robustness. One step toward making AI more robust is to leverage expert knowledge. For example, a farmer/forester in the loop can often bring in experience and conceptual understanding to the AI pipeline—no AI can do this. Consequently, human-centered AI (HCAI) is a combination of “artificial intelligence” and “natural intelligence” to empower, amplify, and augment human performance, rather than replace people. To achieve practical success of HCAI in agriculture and forestry, this article identifies three important frontier research areas: (1) intelligent information fusion; (2) robotics and embodied intelligence; and (3) augmentation, explanation, and verification for trusted decision support. This goal will also require an agile, human-centered design approach for three generations (G). G1: Enabling easily realizable applications through immediate deployment of existing technology. G2: Medium-term modification of existing technology. G3: Advanced adaptation and evolution beyond state-of-the-art.

## 1. Introduction

Cyber-physical systems (CPS), robotics, sensors, data management in general, and artificial intelligence (AI) and machine learning (ML) methods in particular, will significantly change process chains in agriculture and forestry.

Digital transformation in future smart agriculture and forestry requires a human-centered AI approach that incorporates sociological, ethical, and legal issues. Natural intelligence should be augmented—not replaced—by artificial intelligence, like “power steering for the brain”. This is where the human-in-the-loop approach comes in, because this approach incorporates the human experience, prior knowledge, and conceptual understanding of human experts to augment, enhance, and strengthen human capabilities with AI—rather than replacing humans.

In this paper, we first justify why agriculture and forestry are among the most important application areas for all humanity. We then provide definitions of AI and HCAI to facilitate a common understanding, and describe the three main paradigms of ML (supervised learning, unsupervised learning, reinforcement learning) to provide a good introduction for the interested layperson. We then describe the state-of-the-art in autonomous, automated, assisted, and augmented AI systems, giving examples from agriculture and forestry for each classification. Then, in the main body, we introduce three pioneering research areas, namely, (1) intelligent sensor information fusion, (2) robotics and embodied intelligence, and (3) augmentation, explanation, and verification. Finally, we summarize the core ideas of human-centered AI and present two examples: the farmer-in-the-loop and the forester-in-the-loop.

### 1.1. Why Both Agriculture and Forestry Are Important

**Figure 1:**
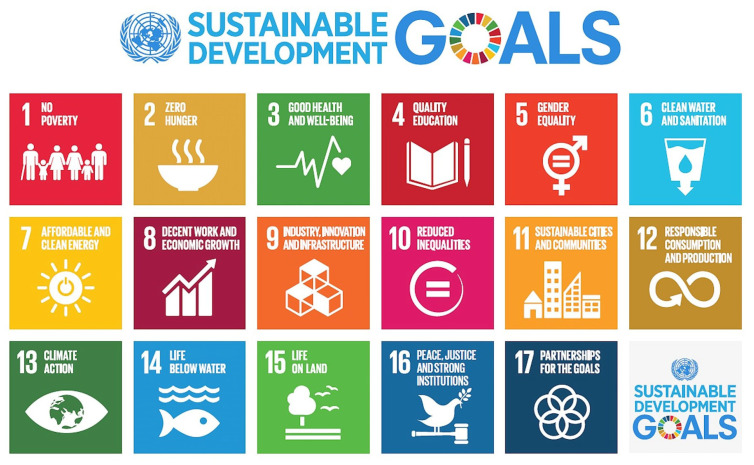
The United Nation’s seventeen Sustainable Development Goals (SDGs) [2].

*When you eat, it is about farming; when you breathe, it is about forestry.* Both agriculture and forestry are vital to everyone on our planet. Both sectors have long and complex business process chains, and in no other sector could new AI technologies contribute more in the future to achieving the Sustainable Development Goals [1] (see Figure 1).

Sustainable development can be seen as the intersection of the goals assigned to the ecological, economic, and social systems. In this context, the systems are interrelated, and simply maximizing the goals for one system does not lead to sustainability; i.e., the impacts of all systems in their entirety must be considered to achieve sustainability.

Barbier and Burgess (2017) [3] note that focusing only on the goals of one system can have consequences for the other systems. Furthermore, they describe sustainable development as a process of tradeoffs between systems. For example, maximizing profits may be efficient for the economic system but threaten biological productivity and biodiversity through environmental degradation. Moreover, the digital transformation process involves many more aspects than just the above systems and their goals. The interactions between systems and actors must always be taken into account.

The European Union (EU) has therefore set out strategies to improve sustainability and meet the Sustainable Development Goals (SDGs). One of the main objectives in this regard is to fully connect farmers and rural areas to the digital economy EC:2017:FoodFarming. With this in mind, the European Commission overhauled the existing Common Agricultural Policy (CAP) to achieve a smarter, modern, and sustainable future for food and agriculture. This resulted in the Green Deal, which is part of the Commission’s strategy to implement the Sustainable Development Goals. The Green Deal also introduced the Farm to Fork strategy, which aims to transform the way food is produced and consumed in the EU. It aims to make the food system healthy, fair, and environmentally friendly.

In addition, recital 22 of Regulation (EU) 2021/1119 [4] and the fact sheet [5] state that the Commission will promote new green business models to reward land managers for reducing gas emissions in general and cutting carbon emissions in particular. This provides positive reinforcement for all land managers regarding the shift to green business models.

Last but not least, the vast amounts of data collected through “smart agriculture” will help farmers farm more efficiently and conserve natural resources [6]. As a result, food will be produced in a more sustainable and environmentally friendly way. The same principle applies to forestry. If the forest can be used in a more sustainable way, allowing not only the trees but also the soil and vegetation itself to regenerate, carbon reduction can be maximized.

The analysis, evaluation, and optimization of sustainable and resource-conserving production processes in agriculture require suitable methods that map the diversity of processes, represent the temporal and spatial variability, and make processes technically controllable. Life cycle assessment (LCA) methods are already available for this purpose. In the future, these methods will also be supported by AI, but let us first take a look at what AI actually is.

### 1.2. Artificial Intelligence

Artificial intelligence is one of the oldest fields of computer science and was extremely popular in its early days in the 1950s. However, the requirements quickly reached the limits of the computing power of digital computer systems at the time. This made AI interesting in theory but a failure practically and especially economically, which inevitably led to a decline in interest in AI in the 1980s. AI only became very popular again a decade ago, driven by the tremendous successes of data-driven statistical machine learning.

Artificial neural networks have their origins in the artificial neurons [7] developed by McCulloch and Pitts (1943). Today’s neural networks consist of very many layers and have an enormous number of connections, and use a special form of compositionality in which features in one layer are combined in many different ways to produce more abstract features in the next layer [8]. The success of such AI, referred to as “deep learning”, has only been made possible by the computing power available today. The increasing complexity of such deep learning models has naturally led to drawbacks and new problems in the comprehensibility of results. This lack of comprehensibility can be very important, especially when using such AI systems in areas that affect human life [9].

Many fundamental AI concepts date back to the middle of the last century. Their current success is actually based on a combination of three factors: (1) powerful, low-cost, and available digital computing technology, (2) scalable statistical machine learning methods (e.g., deep learning), and (3) the explosive growth of available datasets.

To date, AI has reached levels of popularity and maturity that have let it permeate nearly all industries and application areas, and it is the main driver of the current digital transformation—due to its undeniable potential to benefit humanity and the environment. AI can definitely help find new solutions to our society’s most pressing challenges in virtually all areas of life: from agriculture and forest ecosystems, which affect our entire planet, to the health of every individual.

For all its benefits, the large-scale adoption of AI technologies also holds enormous and unimagined potential for new, unforeseen threats. Therefore, all stakeholders—governments, policymakers, and industry—along with academia, must ensure that AI is developed with knowledge and consideration of these potential threats. The security, traceability, transparency, explainability, validity, and verifiability of AI applications must always be ensured at all times [9]. However, how is AI actually defined now, what is trustworthy AI, and what is human-centered AI?

*AI* is difficult to define because the term “intelligence” itself is difficult to define [10]. We therefore follow a very general and widely accepted definition in this paper, namely, the one that describes AI simply as *“automation of intelligent behavior”.* For more details, refer to Russel and Norvig (2020) [11].

For trustworthy AI, it is imperative to include ethical and legal aspects, which is a cross-disciplinary goal, because all trusted AI solutions must be not only ethically responsible but also legally compliant [12]. Dimensions of trustworthiness for AI include: security, safety, fairness, accountability (traceability, replicability), auditability (verifiability, checkability), and most importantly, *robustness and explainability*; see [13].

*Human-centered AI* we define as a synergistic approach to align AI solutions with human values, ethical principles, and legal requirements to ensure safety and security, enabling trustworthy AI. This HCAI concept is now widely supported by renowned institutions (Stanford Human-Centered AI Institute, Berkeley Center for Human-Compatible AI, Cambridge Leverhulme Center for the Future of Intelligence, Chicago Human AI Lab, Utrecht Human-centered AI, Sydney Human-Centered AI Lab) and world-leading experts, such as Ben Shneiderman, Fei Fei Li, Joseph A. Konstan, Stephen Yang, and Christopher Manning, to name a few [14].

The inclusion of a human-in-the-loop in interactive machine learning [15] is thereby not only helpful to increase the performances of AI algorithms, but also highly desirable to counter the earlier fears and anxieties that artificial intelligence automates everything, replaces and displaces humans, and pushes them into passive roles [16].

In addition, integrating a human-in-the-loop (expert-in-the-loop) has many other advantages: Human experts excel at certain tasks by thinking multimodally and embedding new information in a conceptual knowledge space shaped by individual experience and prior knowledge. Farmers and foresters can build on an enormous amount of prior knowledge. Our guiding principle, therefore, is that using conceptual knowledge as a guiding model of reality can help develop more robust, interpretable, and less biased AI models [17]. This can (a) provide advanced contributions to the international research community, (b) find new applications in various AI solutions, and (c) help add value to real-world applications, especially in areas that impact human life (agriculture, forestry, climate, health).

For all these approaches, machine learning is the “workhorse”, and here we are in the fortunate position that the term machine learning is very definable, unlike the term AI. Since a basic understanding is very important here, we will discuss it in more detail in the following chapter.

### 1.3. Machine Learning—The Workhorse of AI

It is important to note that the historical origin of the current successful statistical machine learning lies in the foundations described by Pierre Simon de Laplace (1781) [18], inspired by the work of Thomas Bayes (1763) [19].

Let us consider *n* data contained in a set $D=x_{1:n}=\left\{x_{1},x_{2},\ldots ,x_{n}\right\}$. Let be the likelihood p(D|θ), and specify a prior p(θ). Consequently, we can compute the posterior:

$$p\left(\theta |D\right)=\frac{p(D|\theta )*p(\theta )}{p(D)}$$

The inverse probability allows us to learn from data, infer unknowns, and make predictions [20]. Here is the entry point where a human-in-the-loop can already help: in *defining the prior.* A human-in-the-loop is therefore invaluable, because an expert with many years of experience has this “Bayesian estimate” ready in a fraction of a second in certain situations [21,22,23].

The question of *“where the prior comes from”* and the mere assumption of the same has been a point of attack for frequentist (or classical) statisticians, the opponents of the Bayesian approach [24]. This skepticism enormously delayed the development of “Bayesian” machine learning. It was only through convincing performances in practical applications, particularly in the area of deep learning, that statistical machine learning was finally not only accepted, but gained popularity as the workhorse of AI today [25,26,27].

The field of machine learning began seven decades ago with the idea of developing algorithms that could automatically learn from data to gain knowledge from experience and incrementally improve their learning behavior. It is one of the fastest growing fields at the nexus of computer science and statistics, and at the center of artificial intelligence and data science. Today, the best practical implementations are autonomous vehicles, recommendation systems, and natural language understanding [28]. Ultimately, to reach a level of “usable intelligence”, we need (1) to learn from data, (2) to extract knowledge and new insights, (3) to generalize ideally from a small number of examples (4), to fight the curse of dimensionality, and (5) to disentangle underlying explanatory factors of the data, the causal factors—i.e., to make sense of the data in the context of an application domain [29]. Machine learning algorithms are typically divided into three main categories: (1) supervised, (2) unsupervised, and (3) reinforcement learning:

*Supervised learning* includes algorithms that learn from human-labeled data, e.g., support vector machines (SVM), logistic regression, naive Bayes, random forests, and decision trees [30]. A typical example is a model that receives as input a set of images of crops and a set of images of weeds. Each image is labeled by a human, and the neural network’s task is to learn the features that help distinguish between these two classes [31], which is nowadays performed by neural networks (deep learning approaches) using semi-supervised learning [32]. Typically, modern neural networks decompose inputs into lower-level image concepts, such as lines and curves, and then assemble them into larger structures to internally represent what distinguishes the two (or more) types of images they encounter during their learning process [33]. A trained network that has performed well on one classification task is expected to classify similar inputs (new images of cats and cars) with acceptable success; on the other hand, it is not expected to perform well on other tasks, so the generalization problem mentioned above shows the limits of the capacities of the algorithm.
*Unsupervised learning* includes algorithms that do not require human labels. However, of course, some assumptions are always made about the structure of the data, typically after a visualization process [34,35]. Clustering algorithms that categorize data based on their intrinsic features were the first examples of unsupervised machine learning algorithms. More recent neural network architectures, called autoencoders, compute more compact representations of the input data in a space of lower dimensions than that to which the input belongs. These models can be easily used as generative models after the convergence of the learning process, thereby demonstrating their generalization ability in a constructive way. Even if the human has no direct influence on these algorithms, there is interference in the form of configuration of some meta-parameters; even the choice of the appropriate unsupervised algorithm is made by the designer. Some basic knowledge of the data is required, and techniques such as cross-validation are used to find good parameters and avoid over-fitting problems.
*Reinforcement learning* follows a very different paradigm. The RL algorithm (often called agent here) bases its decisions on both the data and the human input, but the human input is not as direct as labeling [36,37]. To better understand this, it is important to remember that the tasks performed by reinforcement learning algorithms are not about categorizing the input data and learning the meaning of each feature, but rather about learning how to efficiently navigate to a goal. What the algorithm needs to learn is a strategy for reaching the desired state from a starting point, guided not by a predefined plan but by a reward it will receive if it completes either the end goal or key subtasks. The learning process includes several iterations (episodes) in which a so-called agent explores an environment randomly at the beginning and learns from its mistakes, and it eventually finds itself in unprofitable states (from the point of view of the reward). Over time, the agent gathers knowledge about which states are more successful and exploits them to obtain the greatest possible reward. Current state-of-the-art reinforcement learning algorithms are able to find strategies in complex games unknown to humans [38] and also use multiple agents communicating with each other to enable even more efficient strategy discovery [39].

The international ML community aimed from the very beginning to develop algorithms that automatically learn from datasets and improve their performances over time—without a human being involved [40]. This fully automated machine learning (aML)—“press the button and wait for the results”—works well when computational capacity is sufficient and, most importantly, large amounts of training data are available [41], and full autonomy is needed (e.g., in autonomous driving, or in scenarios where humans are absent). The buzzword “big data” is in no way negative, but rather necessary for such automatic learning approaches. However, in some situations we do not have the necessary big data and/or we are faced with complex problems, and in certain applications the use of fully automated approaches is very difficult to realize, e.g., for ethical reasons [42] or legal reasons [43]. Therefore, interactive machine learning (iML) approaches that integrate a human into the loop (e.g., a human kernel [44]), or incorporate a human directly into the machine learning algorithm [45], thereby leveraging human cognitive abilities, are promising approaches. Sometimes (not always, of course) this human-in-the-loop can then bring in human experience, expertise, and conceptual understanding to solve problems that would not be solvable automatically alone, or improve algorithms stuck in suboptimal solutions, not knowing how to improve [46].

## 2. State-of-the-Art AI Technologies

### 2.1. Classification of AI Technologies

If we want to give an overview of AI technologies, it is reasonable to classify them according to degree of autonomy:

- *Autonomous AI systems* that automate decisions without *any* human intervention, e.g., fully autonomous self driving cars [47] and autonomous drones [48].
- *Automated AI systems* that perform labor-intensive tasks requiring certain intelligence and complete them automatically within a certain domain. These have clear goals and tasks. Examples are industrial robotic process automatization [49] and automated forest management [50].
- *Assisted AI systems* that help humans perform repetitive routine tasks faster and both quantitatively and qualitatively better, e.g., ambient assisted smart living [51] and weather forecasting.
- *Augmenting AI systems* that put a human in the loop or at least enable a human to be in control in order to augment human intelligence with machine intelligence and the opposite. Examples range from simple, low-cost augmented reality applications [52] to augmented AI in agriculture [53] and interactive machine teaching concepts [54].

Today, AI can be successfully applied in virtually all application areas [55]. Due to resource conservation and the demand for sustainability, precision concepts, similar to precision medicine, are gaining more attention. These include a very wide range of different information technologies that are already used in many agricultural and forestry operations worldwide [56,57,58,59]. In this context, satellite technology, geographic information systems (GIS), and remote sensing are also very important to improve all functions and services of the agricultural and forestry sectors [60]. Available tools include mobile applications [61], a variety of smart sensors [62], drones (unmanned aerial vehicles, UAVs) [63], cloud computing [64], Internet of Things (IoT) [65], and blockchain technologies [66]. An increasingly important and often underappreciated area is the provision of energy, making alternative low-energy approaches imperative [67].

All of these technologies make it possible to process information about the state of the soil, plants, weather, or animals in a shared network in quasi-real time and make it available for further processes regardless of location. This means that today’s agricultural and forestry systems are being expanded to include other relevant processes/services, and additional datasets are being created for quantitative and qualitative information along the entire value chain for plant production and animal husbandry products and food safety (“from farm to fork”). Let us now show a concrete application example for each type of our four AI classes.

### 2.2. Autonomous AI Systems

“Full automation is one of the hottest topics in AI and could lead to fully driverless cars in less than a decade”, stated a 2015 Nature article [68]. Fully autonomous vehicles are indeed the popular example of AI and are also readily representable, as the Society of Automotive Engineers (SAE) has provided very descriptive definitions for levels of automation in its standards. Levels of automation emerged as a way to represent gradations or categories of autonomy and to distinguish between tasks for machines and tasks for humans. In a very recent paper, however, Hopkins and Schwanen (2021) [69] argued that the current discourse on automated vehicles is underpinned by a technology-centered logic dominated by AI proponents, and point to the benefits of a stronger human-centered perspective.

However, compared to car driving, the complexity of processes in agriculture and forestry is disproportionately higher. Agricultural and forestry systems include virtually all processes for the production of f5 (food, feed, fiber, fire, fuel). In this context, the production processes take place both indoors (buildings and facilities for people, livestock, post-harvest, and machinery) and outdoors, and face much diversity in terms of soil, plants, animals, and people. The temporal resolution of process phenomena varies over an extremely wide range (from milliseconds, e.g., moving machinery, to many years, e.g., growth of trees and changes in soil).

#### 2.2.1. Examples from Agriculture

A major problem in agriculture has always been weed control. In their daily life cycle, plants and weeds compete with each other for soil nutrients, water from the soil, and sunlight. If weeds are left untouched, increased weed growth can affect both crop yields and crop quality. Several studies have already shown that these effects can be significant, ranging from 48 to 71%, depending on the crop [70].

Moreover, in certain cases, crop damage can be so high that the entire yield is not suitable for the market [71]. To prevent this, weed control has emerged as a necessity. Furthermore, with the ever-increasing trends in crop yield production, the demand for process optimization, i.e., reduction of energy losses, herbicide use, and manual labor, is becoming more and more urgent. To meet the above requirements, traditional methods of weed control need to be changed. One of the possible ways to achieve this is to introduce systems that significantly reduce the presence of human labor, the use of herbicides, and mechanical treatment of the soil by focusing only on specific areas where and when intervention is needed. The novel approach based on the above principles is called smart farming or Agriculture 4.0. Moreover, this type of system should involve the performance of agricultural tasks autonomously, i.e., without human intervention, relying entirely on its own systems to collect the data, navigate through the field, detect the plants/weeds, and perform the required operation based on the results of the collected data [72].

These types of systems are known as autonomous agricultural robot systems. Each autonomous agricultural robotic system, e.g., an autonomous robot for weed control, consists of four main systems, i.e., steering/machine vision, weed detection, mapping, and precision weed control [72]. Most agricultural robots are developed for outdoor use, though some of them can operate indoors [73]. Precise navigation of these devices is provided throughout the global navigation satellite systems (GNSS) and real-time kinematics (RTK) [74,75].

However, under certain conditions, localization accuracy may fall below the required thresholds, and then autonomous robotic systems must rely on machine vision and indoor positioning systems, such as adaptive Monte Carlo localization and laser scanners [76]. The above two technologies are widely used and commercially available. Weed control in the row is mainly done by the four conventional weed control methods, i.e., electric, chemical, thermal, and mechanical weed control methods. Currently, weed detection and identification is the most challenging issue. Several studies have addressed this issue, with detection accuracy varying from 60 to 90% under ideal test conditions [72]. Thanks to extensive remote sensing technologies and data processing software, the development of weed maps has become a reality, and together with machine vision, a powerful tool for weed detection.

**Figure 2:**
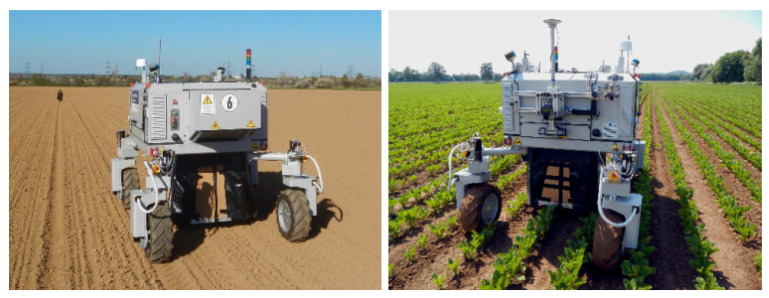
Field robot BoniRob [78].

Some of the most representative autonomous agricultural robotic systems are: The robotic system for weed control in sugar beets developed by Astrand et al. (2002) [77]. The robot consisted of two vision systems used for crop guidance and detection, and a hoe for weed removal. A front camera with two-row detection at 5-meter range and near-infrared filter was used for row detection and navigation, while a second color camera mounted inside the robot was used for weed detection. Initial trials showed that color-based plant detection was feasible and that the robot’s subsystems could functionally work together. The BoniRob autonomous multipurpose robotic platform (see Figure 2) with wavelength-matched illumination system for capturing high-resolution image data was used for weed detection and precision spraying [78], and for ground intervention measurements [79]. Autonomous navigation along crop rows was achieved using 3D laser scans or by using global navigation satellite systems (GNSS). Lamm et al. (2002) developed a robotic system for weed control in cotton fields that is able to distinguish weeds from cotton plants and precisely apply herbicides. A machine vision algorithm was used to determine the diameter of the inscribed leaf circle to identify the plant species. Field tests showed a spray efficiency of 88.8 percent [80].

Similarly to Astrand et al. (2002) [77], Blasco et al. (2002) [81] developed two machine vision system robots for weed control. The machine vision systems are used separately, one for in row navigation and the second one for the weed detection. Precise target weeding was done with an end-effector which emitted electrical charge [81]. Bawden et al. (2017) [82] have developed an autonomous robot platform with a heterogeneous weeding array. The weeding mechanism is based on machine vision for weed detection and classification, together with weeding array which combines precise spraying and hoeing methods for weed destruction [82].

As can be seen, robotic technologies are changing current practices in agricultural technology, particularly in autonomous weed control. The steady increase in research and development in this area will inevitably have a significant impact on traditional agricultural practices.

#### 2.2.2. Examples from Forestry

Timber harvesting is physically demanding and risky, as forest workers often work manually and are exposed to heavy and fast-moving objects such as trees, logs, and dangerous machinery. Over time, timber harvesting has become more mechanized to increase worker safety, productivity, and environmental sustainability.

In the context of increasing productivity through machine use, Ringdahl (2011) [83] found that human operators can become a bottleneck because it is not possible to work as fast as the potential capacity of machines. In trafficable terrain, harvesters and forwarders represent the highest level of mechanization, and they are basically manually controlled by human using joysticks. One way to overcome this human limitation of machine capacity is to change forest working methods in such a way that human activities are reduced to a minimum or are no longer required, like in autonomous vehicles [84]. While autonomous robotic systems are already being used in controlled workspaces such as factories or in simple agricultural environments, the use of autonomous machines in more complex environments, such as forests, is still in the research and development stage. One of the biggest challenges is on-the-fly navigation in the forest.

The most common approach for autonomous navigation in open terrain such as agriculture is based on global navigation satellite systems (GNSS). However, the GNSS signal absorption by the forest canopy leads to position errors of up to 50 m and more, which requires other solutions independent of the GNSS signal [85]. In addition to localization of the forest machine’s own position, the complex terrain and obstacles such as understory, and above all, trees, must also be taken into account when navigating autonomously in forests. In recent years, methods in the field of remote sensing have increasingly been developed to generate digital twins of forests based of terrestrial, airborne, or spaceborne sensor technologies. Gollob et al. (2020) [86] showed that personal laser scanning (PLS) is able to capture and automatically map terrain information, individual tree parameters and entire stands in a highly efficient way. This data can serve as a navigation basis for autonomous forest machines and the optimized operational harvest planning [85].

Rossmann (2010) [85] showed that an initial guess of the forest machine position can be made using an “imprecise” GNSS sensor. 2D laser scanners or stereo cameras on the forest machine (e.g., [87,88,89]) can detect tree positions in the near neighborhood of the machine (local tree pattern). The position of the forest machine can be determined efficiently and precisely by means of tree pattern matching between the stand map (global tree pattern from, e.g., PLS) and the local tree pattern [85]. The initial guess of the machine position with GNSS helps to make the pattern matching more time efficient.

**Figure 3:**
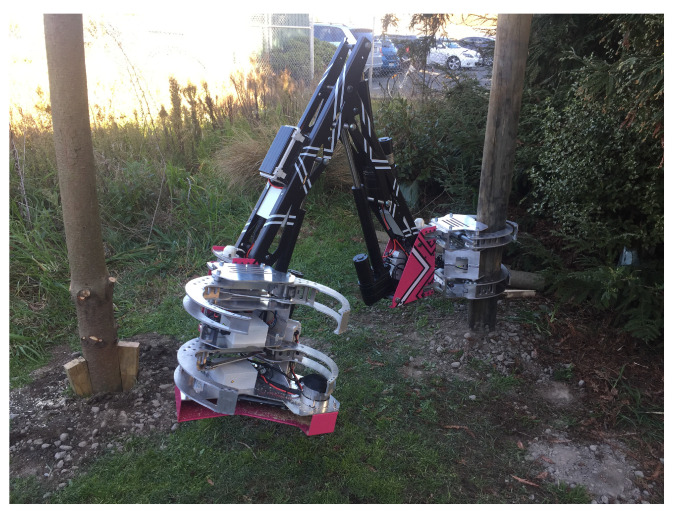
Tree to tree robot (image taken by SCION NZ Forest Research Institute, used with permission from Richard Parker).

Regardless of the challenging navigation, research is also being done on the type of locomotion of autonomous forest machines. Machines that seemed futuristic just a few years ago are already in use or available as prototypes. For example, the concept of animals moving slowly from branch-to-branch was used by New Zealand scientists and engineers to build a tree-to-tree locomotion machine (swinging machine) [84] (see Figure 3). To date, the swinging harvester has been radio-controlled—but with the challenges shown in terms of navigation and sensor fusion, the path to an autonomous, soil-conserving forestry machine is mapped out.

### 2.3. Automated AI Systems

#### 2.3.1. Example from Agriculture

As previously mentioned, autonomous AI systems are relatively advanced and are constantly being developed. These developments and upgrades lead to higher efficiency of the machines. In addition, more and more systems in modern tractors and harvesters are becoming fully automated to minimize the operator’s workload. The two most important domains of automation are situation awareness and process monitoring. For example, machine vision guidance systems are already widely used in modern tractors and harvesters, allowing the machine to automatically align itself with the harvest line without the operator’s help, so that humans can focus on other processes while they do so [90]. Infrared 3D camera systems on harvesters continuously monitor and control bin placement while allowing the operator to focus on the harvesting process [91].

Process monitoring is particularly pronounced in harvesting operations, where speed must be constantly controlled and adjusted according to the operation being performed [92]. The precise application of fertilizers and herbicides is also usually monitored and controlled automatically throughout the process. For this purpose, data from a global navigation satellite system (GNSS) as a guidance system with real-time sensor technology (e.g., Claas Crop Sensor [93]) are communicated among the tractor, the application device, and the task controller via the terminal, which has been done for some time [94].

#### 2.3.2. Examples from Forestry

Cable-yarding technologies are the basis for efficient and safe timber harvesting on steep slopes. To guarantee low harvesting costs and low environmental impacts on remaining trees and soil, the machine position and cable road must be carefully planned. For this planning, usually only imprecise information about the terrain and the forest stands is available. If stand and terrain information were collected with traditional measuring devices such as calipers, hypsometers, and theodolites, these measurements would be labor intensive, time consuming, prone to various errors, and thus limited in their spatial and temporal extent [95].

Thus, the cable road layout is still determined by experts based on rules of thumb and empirical knowledge [96]. Rules for this are formulated, for example, in Heinimann (2003) [97]. Automatic methods (optimization methods) to solve this problem have already been formulated in countries such as the USA and Chile [98,99,100]. However, these optimization or planning methods are largely based on the assumption of clear-cutting and do not use modern sensor technology to capture individual tree and terrain data. For example, high-resolution 3D data in form of a digital twin of the forest combined with well-known optimization functions and expert knowledge is a key factor to optimizing planning of timber harvesting. In this way, automatically optimized cable road planning can help to minimize the environmental impact and the costs for cable yarding (e.g., [96,101]).

In terms of cable yarding, there are also other examples for automation that are already being used in practice: Most cable yarding systems follow a typical scheme (work phases) of unloaded out, accumulate load, loaded in, and drop load on landing. Two of these phases, unloaded and loaded travel, have been automated; thus, the operator can work in the meantime with an integrated processor [88]. Pierzchała et al. (2018) [102] developed a method for automatic recognition of cable yarding work phases by using multiple sensors on the carriage and tower yarder. Further automation steps in cable yarding would be conceivable in the future; for example, the carriage could be equipped with additional orientation sensors, such as laser scanners or stereo cameras, for orientation.

### 2.4. Assisted AI Systems

#### 2.4.1. Example from Agriculture

Assisted AI systems in agriculture are tightly overlapped with automated AI systems. In agricultural applications, machines can independently perform certain repetitive tasks without the human intervention. However, in the decision-making loop, humans are those one who make final decisions [103]. For example, implementation of a wide variety of non-invasive sensors in fruit and vegetable processing, e.g., drying processes, merged together with AI technologies, can be used to control drying processes and changes in shape of vegetables and fruits, and to predict optimum drying process parameters [104]. Several systems, e.g., situation awareness systems such as machine vision guidance systems, though performing their work automatically, can be still manually overridden by an operator [105]. An example is the precision application of fertilizer and pesticides: sprayers can work in fully manual mode [90]. In modern tractors, advanced steering control systems can adjust steering performance in order to suit current conditions, etc. [106]. Furthermore, fuel consumption efficiency can be improved with the above-mentioned technologies [107].

#### 2.4.2. Examples from Forestry

**Figure 4:**
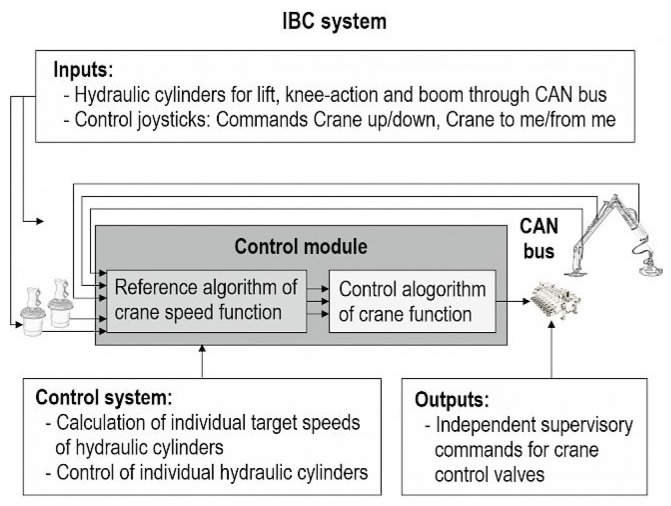
Principles of the IBC system [116].

Operating with forestry cranes requires a lot of knowledge and experience to be productive with a low impact on the environment. Furthermore, trained forestry machine operators are essential for efficient timber production, in particular to reduce harvesting damage to the remaining trees and reduce machine downtime. Ovaskainen et al. (2004) [108] has shown that the productivity of trained harvester (Cut-to-length (CTL)) operators varies by about 40% under similar forest stand conditions. The study hypothesizes that efficiency differences are related to aspects of operator crane experience based on deliberate practice of motor skills, situational awareness, and visual perception. The state-of-the-art in crane control is the use of two analog joysticks, which are controlled by the two hands and/or the fingers. The joysticks provide electrical or hydraulic signals that control the flow rate of the hydraulic system and thus enable precise movement of the individual hydraulic cylinders. Motor learning is the key to smooth crane movements and harvester head control. Forest machinery machinists make approximately 4000 control inputs/h, many of which are repeated again and again but always have to be applied in a targeted manner [109]. Purfürst (2010) [110] found that learning to operate a harvester took on average 9 months. Furthermore, the operator must also master decision making and planning in order to achieve an appropriate level of performance. In summary, it can be stated that harvester/forwarder operation, especially crane operation, is ergonomically, motorically, and cognitively very demanding. To improve this, existing forestry machines are constantly being improved. Modern sensors (e.g., diameter and length measurement) combined with intelligent data processing can help to assist certain operations, such as processing stems into logs by automatically moving the harvester head to predetermined positions depending on the stem shape and log quality. On the one hand, this reduces the workload of the harvester operator, and on the other hand, optimizes the profit on the timber. Other good examples of how intelligent assistance can make work easier and also more efficient for the machine operator are the intelligent crane control systems from John Deere Forestry Oy (IBC: Intelligent Boom Control [111], see Figure 4), Komatsu (Smart Crane [112]), and Palfinger (Smart Control [113]). In such systems, the crane is controlled via the crane tip (harvester head or grapple), and the crane’s movement is automatically done via algorithms. Therefore, the operator is not controlling individual rams, but only needs to concentrate on the crane tip and control it with the joysticks. The system also dampens the movements of the cylinders and stops jerky load thrusts in the end positions, which enables jerk-free operation. The smart control results in less fatigue for the machinist, making them more productive overall. Results from Manner et al. (2019) [114] showed that if the crane is controlled with IBC compared to conventional crane control, the machine working time for the forwarder is 5.2% shorter during loading and 7.9% shorter during unloading. It has already been shown that the use of such a smart crane control system makes it much easier to learn how to operate harvester or forwarder machines [88,115].

### 2.5. Augmenting AI Systems

#### 2.5.1. Example from Agriculture

**Figure 5:**
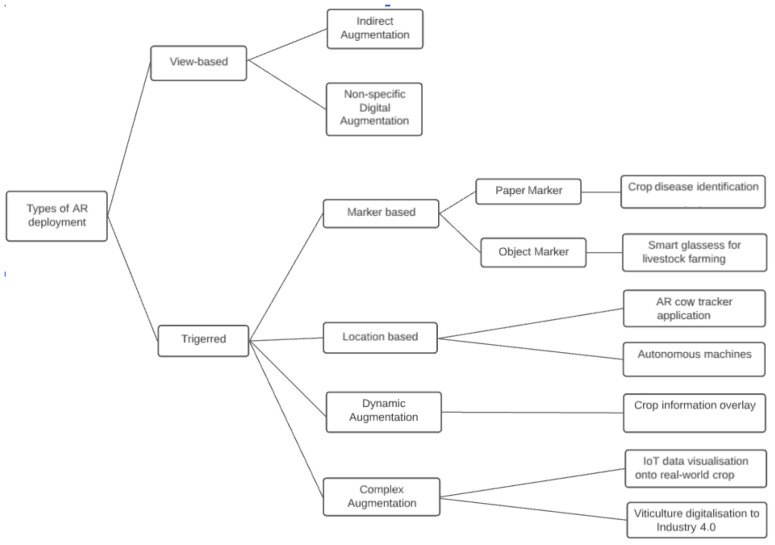
Types of AR deployment within crop and livestock management. Please refer to the excellent overview by Hurst et al. (2021) [117].

**Figure 6:**
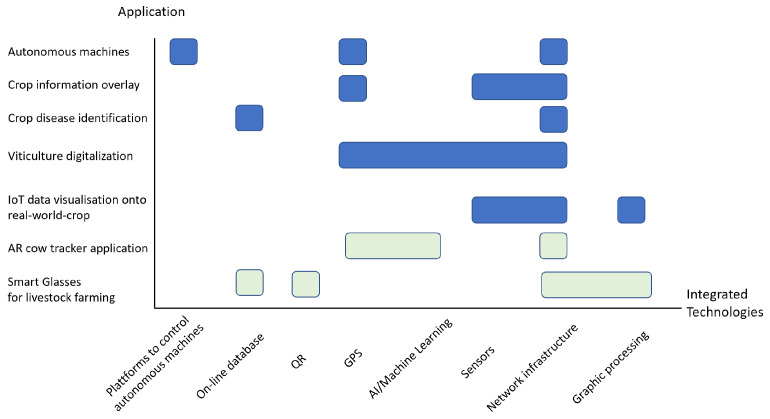
Analysis of technologies coupled with AR in Farming. Dark blue refers to crop-bases articles, whereas light green is for livestock, adapted. For details, please refer to the original paper by Hurst et al. (2021) [117].

A good systematic for AR applications is given by Hurst et al. (2021) [117] (see Figure 5 and Figure 6), which has subdivisions into (1) marker-based, (2) markerless (location-based), (3) dynamic augmentation, and (4) complex augmentation, subdivided by AR type. Based on the four classifications, there are a lot of potential applications of AR in agriculture.

Augmented reality (AR) enables the combination of a real environment and an interactive experience, e.g., synthetically generated information. It is necessary to clearly distinguish augmented reality (AR) from virtual reality (VR). Virtual reality uses a synthetically generated environment to replace the real environment, whereas augmented reality enhances the real environment with synthetically generated data [118]. Both technologies have found many applications in a wide variety of domains [119,120,121]. However, agricultural applications require the technology to be even more user-friendly, for example, by replacing smartphones and tablets with smart glasses [122]. This would provide operators with hands-free capabilities and a less constrained user interface. Although there are still some technical and technological drawbacks, smart glasses have emerged as promising potential candidates for the main platform for AR.

Meanwhile, head-mounted AR displays, for example, are being used to help farmers detect plant diseases [123]. Through the head-mounted display camera that observes the plants, images of the plant leaves are captured in real time and sent to the cloud server for analysis, which also provides a lot of room for detecting defects/anomalies. After post-processing the data in the cloud, the results are transmitted back to the head-mounted display. In this way, a augmented reality head-mounted display allows less experienced farmers to inspect the field more efficiently and quickly in search of infected plants. In addition, such technology can also help train and educate the farmer directly in the field. The farmer’s knowledge remains invaluable, as the expert can contribute their domain knowledge to future machine teaching AI solutions.

AR smart glasses are already used for scanning QR codes during characteristic livestock activities such as feeding and milking [124]. The initial results show that the above-mentioned glasses can provide significant help to farmers by enabling real-time consultation, data collection, data sharing, etc., and proved to be a useful tool for herd management and feeding, where many more applications will bring benefits to farmers in the future, especially through AI.

**Figure 7:**
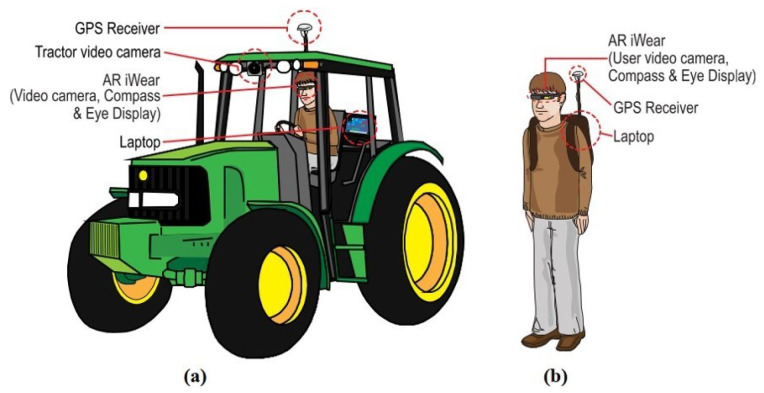
AR-based positioning assist system: (**a**) tractor mounted, (**b**) manual mounted [125].

AR smart glasses (see Figure 7) [125] have already been implemented to create a system that assists the operator during field operations such as plowing and fertilizing. This can minimize the strain on the operator caused by constantly tracking maps, light bars, etc., which can be especially pronounced in large, irregularly shaped fields. With the help of AR glasses, the operator thus obtains data on their trajectory, speed, etc., which is superimposed on the data of the treated surfaces and largely simplifies the operator’s work. It should be mentioned that this system can be used both inside the machine and outside it. This system has proven to be very useful in fertilizing, spraying, and plowing operations.

Besides the above-mentioned examples, augmented reality-related research is on the rise, mainly focusing on greenhouse management [126], weed management [127], and farmer management support [128].

#### 2.5.2. Examples from Forestry

Automated and assisting systems combined with a variety of modern sensors increase the productivity and quality of forest work. Even if we are far from using the full potential of smart technology in forest machines, the operator is already receiving a large amount of information compared to traditional machines. In addition, forestry machines are also increasingly networked with each other (e.g., harvesters and forwarders) and also have possibilities to retrieve or exchange information from the Internet. On the one hand, this abundance of data helps to train autonomous, automatic, or assisting processes; but on the other hand, it leads to a challenge in mediating the information to the operator. However, in addition to many of the benefits of “big data”, negative effects can also occur in the form of divided attention, information overload, and operator stress. A further increase in the information burden on the operator can potentially lead to higher chances of human errors that could harm not only the operator, but also people, machines, and objects in the neighborhood of the machine [129,130,131]. Augmented reality can help to provide the information better and more efficiently to the machine operator. Augmented reality, which matches data generated by sensors and algorithms with the user’s actual perception of the environment, can improve the operator’s awareness of the machine, the environment, and the specific work progress. Sitompul and Wallmyr (2019) [129] defined for forest applications that augmented information could be provided for two types of operation: in-cabin operation and remote operation. Traditionally, heavy machines, such as forestry machines, are operated in the cab. In recent years, there have been repeated efforts and studies to move the operator from working in the cab to working in a remote control station, which is called teleoperation. With regard to cab work, Kymäläinen et al. (2017) [132] and Aromaa et al. (2020) [133] have proposed to show technically related visual obstacles of the forestry machine (e.g., crane) transparently on a display.

The hidden areas (behind the crane) could be seen with the help of cameras. This increases safety for the surrounding area. In addition to classic screens, there is also the option of projecting information directly into the operator’s field of view via a heads-up display. For example, the operator can be provided directly with information about the bucking optimization of the harvester without having to look at a separate monitor [134]. Teleworking mainly leads to hazard minimization for the operator. The biggest challenge in teleworking is sufficient visibility, as the fields of view of the cameras attached to the machine are limited [135]. Furthermore, depth vision is lost due to the most commonly used 2D displays, which makes it difficult to position the crane exactly and grip logs.

**Figure 8:**
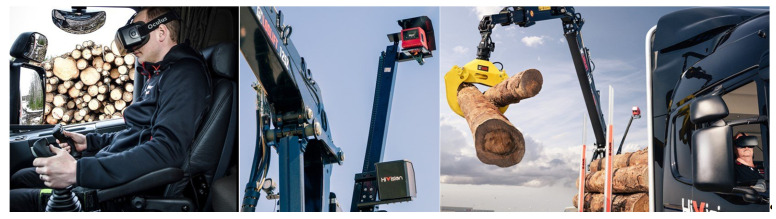
Hiab HiVision VR system [136].

For timber loading onto trucks, Hiab (HiVision [136], see Figure 8) and Palfinger (Virtual Drive [137]) each offer a control system for forestry cranes. It allows the operator to control the crane from the truck cab while monitoring the environment with the help of a camera and VR goggles. The advantages of the system are that the truck driver no longer has to leave the cab, thereby avoiding hazards, and the operator is ergonomically positioned.

Due to the high cost of machinery and the dangerous nature of the work, great importance must be placed on the training of forestry machine operators. Thus, in addition to supporting operational work, augmented reality also offers advantages in education and training. With realistic simulations of forestry machines [138] and forest [139], a student can be trained in a safe environment in all work processes that occur in real operations.

## 3. AI Branches of Future Interest: Frontier Research Areas

**Figure 9:**
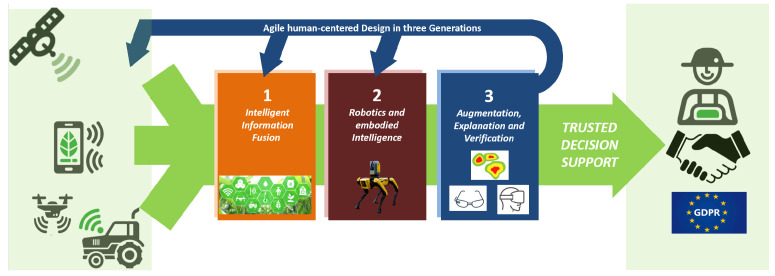
3 × 3: The three frontier research areas with agile human-centered design in three generations. G1: testing existing technology, G2: adapting existing technology, G3: advanced adaptation going beyond state-of-the-art.

To achieve practical success in agriculture and forestry, we identify three important AI frontier research areas (see Figure 9: (1) intelligent sensor information fusion, (2) robotics and embodied intelligence, and (3) augmentation, explanation, and verification technologies for trusted decision support. Such developments need novel, agile, human-centered design approaches with three generations (HCD-3G):

- Generation 1: Enabling an easily realizable application through immediate deployment of existing technology which can be solved at the “bachelor level”.
- Generation 2: Medium-term modification of existing technology, which can be solved at “master level”.
- Generation 3: Advanced adaptation and evolution going beyond state-of-the-art at “doctoral level and beyond”.

In the following chapter we describe some open challenges and future research issues from which we expect three forms of added value to arise: (1) contributions to the international research community, (2) theoretical contributions beyond the state-of-the-art, and (3) practically useful contributions that ultimately add value for users in agriculture and forestry.

### 3.1. Intelligent Sensor Information Fusion

Smart applications in agriculture are characterized by the gathering of different types of data with the use of adequate sensors: soil, plant, animal, and environment sensors are used together for farm monitoring and management purposes [140]. There are a plethora of possibilities with the use of sensors that monitor physical and chemical signals, such as temperature, moisture, pH, and pollutants in real-time. One definition of multi-sensor fusion can be found in [141]; the same phenomenon or entity that is described by an unknown random variable is observed by different types of sensors. Through comparison, aggregation, and combination of several types of sensor data that are error-prone in general, the uncertainty about the random variable is decreased. The sensors, particularly in agricultural and forestry applications, are exposed to hard environmental conditions that greatly vary, and therefore, cannot deliver error-free measurements as the ones in a protected laboratory environment. In every step of the pipeline, including sensor data gathering, transfer, and storage, different types of faults can occur [142,143]. Adequate quality management (QM) measures [144] need to be implemented—both human-supervised and automated—to ensure a reliable outlier analysis and fault detection (FD) [145] as early as possible.

Sensor fusion can help not only error detection but also help the discovery of new insights with a variety of algorithms that basically compare the information content of several types of sensor data [146]. Data are typically combined under particular constraints, and at the same time, several conditions of consistency between them can help artificial intelligence systems to automate data preprocessing to a certain extent. In the work of Lee et al. (2010) [147] both thermal and RGB images were fused on the basis of similarity measures. Beyond raw data fusion, features that characterize the input data can be extracted, fused, and processed by individual AI algorithms, parallel to the ones processing the raw data [148]. The insights from both systems can be then combined in a weighted or voting fashion, which generally provides a better resulting performance, since each part grasps different aspects of the data [149,150]. Clustering methods and hierarchical self-organizing maps (SOM) are also used to detect intercorrelations between the data to enable an even more effective fusion process [151]. In conjunction with novel wearable sensors [152], these can provide tremendous benefits for smart farming and smart forestry, providing important information to optimize plant growth, combat biotic and abiotic stressors, and increase crop yields.

Beyond sensors, there are also other sources where data can be gathered, such as robots, handheld devices, drones, airborne laser scan data, and weather satellite data; we see enormous added value for a wide range of applications. Machine learning is an excellent way to achieve sensor fusion effectively. A detailed review of state-of-the-art methods can be found in [153]. Least squares support vector machine (SVM) classifiers [34] (see also Section 1.3 about supervised learning) were some of the first that dealt with different types of agricultural data that might have different ranges and be sampled at completely different timepoints.

- Open Challenge G1: “Garbage in-garbage out”—this motto of data scientists is there to always reminds us of the fundamental property of information entropy, as defined by Claude Shannon [154,155]. Since the information that is lost can never be fully and perfectly reconstructed—in the best case only approximated—one of the first and foremost goals is to make sure that the sensor information is gathered with as few problems as possible. The sensor’s data gathering capability in difficult environmental conditions, and the continuous, (near-)real-time operation thereof and for the servers, need to be ensured. The quality and the sensitivity of the sensor are defined by production but need to be verified in practice. Together with human experts, the gathered data (both sensors and satellite) need to be analyzed, and the individual characteristics thereof need to be specified.
- Open Challenge G2: Human experts have the capability to recognize abnormal behavior in sensor data by adequate visualizations and statistics. Fault detection software, on the other hand, has to rely on anomalies in the data, which means that (1) the outliers need to be “rare” (relative) and (2) the values of different sensors need to be compared with each other. What the vast majority of the sensors will track, will be roughly decisive for describing the whole data gathering, preprocessing, transferring, and detecting a fault either in a few of the sensors, all of them, or even the whole data transfer process.
- Open Challenge G3: A reliable real-time data fusion system that has the intelligence to know when it is advisable to fuse different sensor data. If data from the majority of different sensor sources are inconsistent, then the data fusion process should rather be discarded, since this is an indication of a fault in the data pipeline. Ensuring that the gathered data lie in an acceptable range, are consistent with each other, and obey roughly some expected physical rules (radiance, humidity with respect to temperature) can open the path for successful and insightful information fusion.

### 3.2. Robotics and Embodied Intelligence

Pieter Abbeel puts it in a nutshell in his presentations, e.g., at CVPR 2021: *The hardware in robotics is there, it is capable and usable. The real challenge is that the robots lack intelligence-which is defined by the underlying software. This motivates why we do not focus on hardware aspects, but take existing hardware and work on “bringing the intelligence into it”.*

Combining human capabilities with robotics and embodied intelligence is a promising approach, which is still largely unexplored and has enormous potential. The team-ups of robots and humans could lead to exceeding their individual performances by combining the robot’s motor, vision, and computation capabilities with the perceptiveness and deep understanding of humans. To achieve this goal, several current approaches have to be built upon and combined.

For one, it is essential to bring RL agents up to a certain base performance level before their human interaction should begin, in order to allow efficient use of humans’ time and to avoid frustrations. Agents can be trained with synthesized or already gathered data under simulated conditions. Of course, deviations from real conditions need to be expected, but nevertheless, the simulations can provide an upper limit in expectations and also help define some minimal requirements for the on-site operation.

To recognize if the agent has reached this acceptable base level, and since many RL problems are currently solved by deep RL techniques [37,156,157], performance metrics and explainable AI (XAI) techniques can be used [158]. These techniques can demonstrate the underlying reasons for the decision-making process of a deep RL agent and help humans understand which parts of the current state were most relevant for the action. Furthermore, they help humans to understand the agent’s overall strategy and answer “what if” questions [17]. Then, the robot can efficiently learn through interaction with a human either by human demonstrations or preference-based learning [159]. Ultimately, the ongoing team-up can benefit from active learning approaches were edge cases are caught and solved by the human—also with the use of XAI methods—helping the robot to learn from those unknowns and generalize better in the future.

In order to enable a successful and efficient interaction between humans and RL agents, models need to have sufficient cognitive capacities for judging and further improving their behavior. A good example for this requirement is preference-based learning [159].

In preference-based learning, a robot presents two options (such as movement policies) to a human operator, who selects a preference and with that facilitates the challenging reward-selection process. In order for this to be effective, it is beneficial if the robot presents two “meaningful”, coherent movement policies, instead of the jittery behavior seen in newly instantiated models [160]. This exemplifies a more general statement: it is easier to judge the consistency of an already trained model, because it has already developed past the initial noise of random initialization.

One approach is pre-training, where models learn to reconstruct images in mostly self-supervised fashion from masked inputs. A drawback on this approach is that the model inevitable learns on the bias and noise of the given samples, which hampers the goal of learning noise-invariant concepts [161]. Contrastive pre-training extends this approach by masking or modifying (cropped, rotated, colored etc.) the samples and then reconstructing them, adding noise in the training process and leading to a more robust generalization. A concrete example for this application is the combination of contrastive pre-training and data-augmented RL, as proposed by Srinivas et al. [162]. To ensure that agents can benefit from this pre-training, the exploration–exploitation dilemma has to be tackled. Especially when training in an unsupervised fashion, the agent can get stuck in the exploitation phase without having sufficiently explored the available states. Liu and Abbeel [163] proposed a state-entropy reward to ensure a better state-coverage with unsupervised pre-training.

A second approach is to transfer knowledge between networks, before fine-tuning them for a certain task. Xu et al. [164] showed this approach by quickly learning knowledge (offline) from task-specific teachers, before continuing with an online-learning approach to further improve results. This also exemplifies how a network is brought up to speed, before being further trained in a continuous, online fashion.

A central challenge in reinforcement learning is finding a good goal function, which can be extremely laborious and still fail to consistently capture the intended goal [160]. To work around the task of explicitly stating the goal function, demonstration learning leverages the demonstrations of an exemplary solution for a task by a human to teach the RL agent. The agent can then extract behavioral priorities (capabilities) by fitting generative models to a large offline dataset of demonstrations. This, however, often requires a large dataset of samples to achieve good results. This approach was improved upon by Lin et al. [165], who used sample-efficient demonstrations to help agents to better explore during training and derive the rewards from the given demonstration.

**Figure 10:**
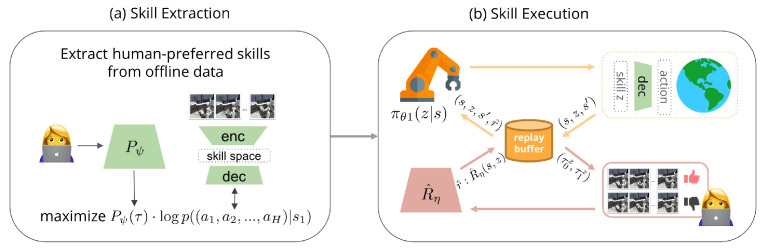
Skill preferences (SkiP) [166] consists of two phases. During the skill extraction phase, human feedback is used to learn skills. During the skill execution phase, human feedback is used to finetune the skills to solve various downstream tasks. First, skills are extracted from a noisy offline dataset with human feedback to denoise behavioral priors. Second, skills are executed with RL in the environment with task-specific human feedback.

However, a drawback to demonstration-based generative models is that they inherit perturbations in the raw data, and therefore can gain unusable skills. To better match skill extraction to human intentions, Pieter Abbeel’s group recently introduced the so-called skill preferences (SkiP) approach, an algorithm that learns a model about human preferences. After extracting human-preferred skills, SkiP also uses human feedback to solve downstream tasks with reinforcement learning. This has recently been used to solve complex, multi-step manipulation tasks [166]; see Figure 10.

The general idea of “learning behavioral priors with human feedback” (skill extraction; see (a) in Figure 10) is to use human preferences in order to fit a weighted behavioral prior over an offline dataset of (potentially noisy) demonstrations. SkiP builds on prior work for behavioral extraction from offline data via an expected maximum likelihood latent variable, e.g., as done in the OPAL approach [167]. They considered a parametric generative model $p_{alpha}\left({actionseq}_{t}|s_{t}\right)$ over action sequences where:

$${actionseq}_{t}=\left(a_{t},\ldots ,a_{t+H-1}\right),$$

which represents a behavioral prior, which was trained to replicate the transition statistics in the offline dataset:

(1) $$p_{\alpha }\;\in \;\arg\max_{\alpha }\;E_{\tau ∼D}\left[\sum_{t=0}\log\left(p_{\alpha }\left(a_{t}|s_{t}\right)\right)\right].$$

A third approach we want to highlight is active learning, where the agent actively queries the user for unknown data points, and is often guided by heuristics to determine uncertainty for sample selection. This has proven to be an efficient method for training semi-supervised models that has far lower costs. Fang et al. [168] showed an example for such an active learning approach for deep learning where they frame the sample selection process as an RL problem and with that generate a transferable heuristic. Rudovic et al. [169] showed how the active learning approach can also be used for fine-tuning by quickly personalizing classifiers on multi-modal user data.

**Figure 11:**
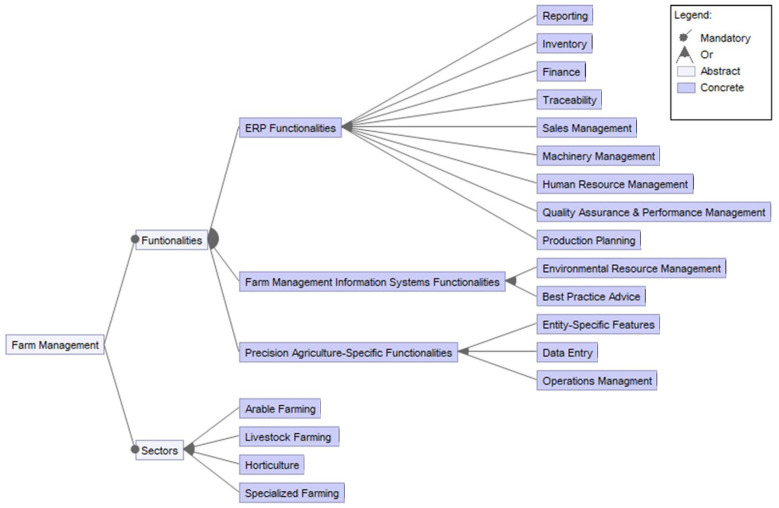
A feature model for precision agriculture farm management [172].

There are many farm management and information systems (FMIS) available on the market. What they virtually all have in common is not mapping an entire farm, although terms such as “digital shadow” or “digital twin” can increasingly be found in the literature (e.g., [170,171]). To date, only so-called isolated solutions can be assumed. Future challenge lies in the complete mapping of entire farm systems, including the merging of all data streams in order to obtain information for the optimization of individual processes and entire farm systems. In addition, it must be made possible for the farmer to incorporate their expert knowledge into these systems in order to make the individualization of a virtual farm operating system a reality, as is shown by Groeneveld et al. (2021) [172] (see Figure 11). One of the research questions is how to include the expert-in-the-loop in the most efficient way.

Throughout the last decades, a wide variety of autonomous agricultural vehicles have been developed, based on different platforms, control units, sensor sets, operational algorithms, etc. [173]. Throughout the careful observation of all of these autonomous agricultural robots, several conclusions which can be used as a guidance for the designers can be derived. First of all, the most practical transmission system for the wide variety of agricultural autonomous vehicles is automatic transmission. Automatic transmission allows us precise control of performed operations, e.g., harvesting and fertilizing [73]. In order to control processes performed by autonomous agricultural vehicles, each vehicle has to have access to the engine controlling unit. Furthermore, each autonomous vehicle must have a computer which receives and sends commands from/to the sensor set, and send commands to actuators. Navigation is mainly performed by GNSS-based systems. Last, but not the least, controlling algorithms should be as simple as possible [174].

We propose several research directions that are beneficial for successful human–machine interaction in the scope of human-centered AI in embodied intelligence. A fundamental aspect that has to be taken into account is that generating trust in the system is essential for a fruitful interaction between the end-user and the robot. This trust is built by making the end-user understand the perception and decision-making process of the robot agent, which necessitates a focus on explainability.

Current explainability research, however, focuses mostly on explanations for experts and system developers, less on the end user [175]. We suggest that further research is required into what human (and end-user)-centered AI systems could look like, focusing on aspects such as real-time computation, understandability, explanations for lay persons, and predictability of robot behavior in general. First-generation tasks can focus on evaluating how current xAI approaches work and generating requirements for explainability from an end-user perspective. Second-generation tasks could then focus on testing and evaluating different approaches, which provide better explainability within the given constraints. The high-level, third-generation approach is developing a coherent framework for requirements and solutions providing explainability for embodied intelligence, collecting and exposing the developed xAI solutions. This approach also encompasses guiding the development of approaches in the second generation, ensuring an even coverage of this multi-faceted explainability challenge.

Such a process eventually allows us to provide a comprehensive set of tools that developers of robotic systems could easily use for facilitating their human–robot interactions with strong explainability approaches.

- Open Challenge G1: Before even starting on-site operations, several experiments with synthetic data under simulated conditions must be completed. The scope of the challenges needs to be documented, along with the basic elements of the RL problems that will be faced down the line. Basic thoughts about the state and action space of the problems at hand, the reward strategy, the obstacles, and the limitations need to be examined. The feasibility of the solution, its scope, computational resources, and time resources need to be defined. The first simple prototypes in the laboratory that use deep RL need to work efficiently.
- Open Challenge G2: The next level must incorporate stronger modeling testing, where both the agent and the environment’s characteristics will be represented by state-of-the-art AI software. The robot’s behavior has to take into account that noisy data and edge cases are things that will be encountered. Gradually, it has to move on from the more idealized case of Challenge G1 to real-world data that contain faults, have drifts, and are representative of an environment that is much more complex. This is planned to be an incremental process that will step-wise make the agent capable of dealing with real, on-site situations. The decision-making process of the robot, while confronted with newer, more complex situations, must also be highlighted through XAI methods. By that means, human experts can control if the robot is following plausible principles or relies on Clever–Hans correlations [176].
- Open Challenge G3: The milestone goal of embodied intelligence for agricultural applications will be for the robots being able to perform the required tasks in real conditions that are far more complex than the ones encountered in the simulations of Challenge G1. At this level, the robot is well capable of recognizing when it can act autonomously, providing an understandable explanation for its decisions to the human with the help of XAI, learning from human feedback, and also enhancing the human’s expert knowledge—since the robot might discover a new solution to the encountered problems, as in the game of Go [38].

### 3.3. Augmentation, Explanation, and Verification Technologies

Visualization methods and augmented reality (AR) are also becoming more sophisticated thanks to advances in AI. What is essential, however, is that these technologies are now very widespread and affordable, and are therefore used in a wide variety of application situations as tools for decision support [177]. Domain experts can be provided with contextual and relevant information in an unobtrusive way (AR glasses for forestry technology). In this context, a new technology trend is very important: *situated visualization (SV)*, which can be seen as the presentation of information in its semantic context [178].

However, the presented data need to be filtered, to not overwhelm the user. In order to archive the best possible visualization for a user, Julier et al. (2000) [179] proposed an approach of information filtering whereby the information is prioritized according to the user’s needs. In addition, the user should be able to interact with the presented data. The full potential of this interaction can be exploited with the use of AR. For example, the data could be automatically filtered using the user’s context [180]. Moreover, the AR experience could be enhanced by drawing the user’s context from certain objects the user interacts with in the physical world.

AR technologies are very affordable and widespread, due to the fact that smartphones and tablets can be used as mobile displays. Head-mounted see-through displays, such as the Microsoft HoloLens and Google Glass, provide direct observation of the physical world while displaying virtual objects. They are easy to use, lightweight, and even allow the user to operate with both hands.

Google Glass is based on Android, and therefore allows the user to install applications from the Google Play Store [181]. The new Google Glass Enterprise Edition 2 allows the wearer to communicate through video with other people and let them experience the viewpoint of the wearer [182]. Furthermore, AR can be used as a tool for SV, whereby the information is visualized close to the location of the physical object [183].

Another effective way to approach information filtering is to use AI software for real-time anomaly and fault detection [145]. Farmers and foresters are mostly interested in being informed about abnormalities in their everyday working process so that they can intervene as soon as possible. This can be gradually done with the use of intelligent AI software that uses the knowledge of the domain expert and the available data to define the characteristics of normal operation vs. faulty case [184,185]. Information fusion is going to be incredibly beneficial, since a faulty state is usually characterized by anomalous behavior on several data sources almost at the same time. After a period where such a fault detection (FD) system has proven its reliability and correctness, concentrating on prioritizing data leading up to and indicating the fault will enormously help information filtering.

- Open Challenge G1: Create the first visualizations for multi-modal data gathered from the sensors. Concentrate only on the adequate presentation of the data to domain experts and consider some usability aspects, without consideration or software development of data filtering in mind. Incorporate principles from graphical design and use all the facilities that one can ideally have, such as big monitors, Google Glass, and augmented reality systems (AR).
- Open Challenge G2: Work in collaboration with human experts (forester and farmer) and see what are their principal needs, requirements, and expectations from an AI solution. Define with their help what their priorities are and what characteristics of the data differentiate between normal vs. abnormal behavior. Use the results from Challenge G1 to show them static visualizations from particular situations, and let them pinpoint, choose, and enhance those visualizations. Define with them use-cases that take into account which information is mist important for their decision-making process and how to prioritize and present it so that they are informed precisely, as fast as possible, but without being overwhelmed.
- Open Challenge G3: Develop a real-time fault detection AI solution that encompasses all parts of the pipeline: (1) Data gathering, preprocessing, and fusion, (2) adequate visualization as implemented for Challenge G2 but now real-time, and (3) real-time fault detection with the use of efficient and explainable AI solutions. This pipeline is not static, since neither the human nor the AI software is perfect, nor can either prepare itself for every possible fault and condition that might occur. The components adapt to new anomalies, user requirements, domain-expert knowledge, and challenges that will arise from the use of more data, more sophisticated XAI methods, and quality management techniques.

## 4. Human-Centered AI and the Human in the Loop

A human-centered AI approach seeks to promote the robustness of AI algorithms by incorporating a human in the loop, and advocates a synergistic approach to allow humans to control AI and to align AI with human values, ethics, and legal requirements to ensure privacy, security, and safety [12].

### 4.1. Interactive Machine Learning with the “Human in the Loop”

The central challenges of real-world AI applications are in the uncertainty of the data—data can be missing, noisy, dirty, unwanted, etc.—and most of all, many problems in the real world are computationally hard, which makes the application of fully automated approaches difficult or even impossible, or at least the quality of results from automatic approaches might be questionable. Most of all, the complexity of sophisticated machine learning algorithms has detained non-experts from the application of such solutions.

The integration of the knowledge of a domain expert can sometimes be indispensable, and the interaction of a domain expert with the data would greatly enhance the whole machine learning process pipeline.

**A human expert can (sometimes—not always, of course) bring experience, knowledge, and contextual understanding into the machine learning pipeline, which is invaluable to understanding and solving problems from our everyday world. This is what our best AI algorithms lack to date.**

Hence, *interactive* machine learning (iML) puts the “human in the loop” to enable what neither a human nor a computer could do on their own. This idea is supported by a synergistic combination of methodologies of two areas that offer ideal conditions towards unraveling such problems, human–computer interaction (HCI) and knowledge discovery/data mining (KDD), with the goal of supporting human intelligence with machine intelligence to discover novel, previously unknown insights into data (HCI-KDD approach [186]).

**The human-in-the-loop approach is defined as algorithms that can interact with*****both computational agents and human agents*****and can optimize their learning behavior through these interactions.** [45].

### 4.2. Human-Centered Design

Human tasks change from time to time, and so do their requirements. In order to compromise on the vast demand for AI while providing users with the best possible experience, the design of AI technologies and interfaces is crucial. Holzinger et al. (2022) [187] showed the continuous changes in users’ profiles and requirements. The use of AI technologies illustrated that some users might want to try new technologies, whereas others might not. Several different reasons can influence the users’ trust, and therefore the possible usage of certain systems. Hence, it is necessary to get to know the potential users and keep their knowledge, experience, and goals in mind when designing systems. For example, a farmer will most certainly be interested in different data than a machine learning expert. They might use technology based on the same model, but they pursue different goals, and therefore desire different information or visualizations. Agile software development methods, particularly agile user centered design methods [188], are increasingly being used in industry, and are ideally suited for our outlined research and development approaches. However, these methods still lack usability awareness in their development lifecycles, and the integration of extreme usability methods into agile methods is necessary [189], which can help to fulfill the approaches of the “augmented farmer” or the “augmented forester”, which is similar to the “augmented pathologist” [190] or the “augmented radiologist” [191].

### 4.3. Farmer-in-the-Loop

The evaluation of the sustainability of a new technology is becoming of vital importance these days. A sustainability assessment (LCSA) includes a life cycle assessment (LCA) for estimating environmental impacts [192]; life cycle costing (LCC) for assessing economic issues [193]; and finally, a social life cycle assessment (SLCA) [194]. With this holistic assessment, processes can be evaluated over their entire life-spans (e.g., production of raw materials, manufacturing, use, end-of life treatment, and recycling and disposal of the product). In addition, based on the results, recommendations regarding support for sustainable development can be provided [195]. In the last few decades, assessments of systems have mostly focused on economic and environmental issues, as methods assessing social sustainability are still under development. Hence, the following paragraph will focus on environmental and economic issues regarding assessing sustainability of AI, specifically the implementation of sensors.

The use of sensors is a common practice in AI applications and robotics alike. Precision agriculture relies on sensors to gather the necessary data to respond to the temporal and spatial variability of crop production. Remote and proximal sensors are the two most common techniques to gather temporal and spatial crop information. Proximal sensor readings to determine crop input requirements started in the 90s [196]. In 1998, the first normalized difference vegetation index (NDV) sensor reading was used to determine the nitrogen requirements in a Bermuda grass field in Oklahoma [197]. Remote sensing, especially the use of satellites to determine spatial nutrients in soil and plants, started in the early 90s [198]. The use of sensors leads to varying the rates of crop input requirements, having positive environmental and economic impacts on crop production. Hotspots such as soil acidification, water eutrophication, and global warming potential (GWP) may be reduced. Profits can be also be achieved by using sensor-based technologies. Several studies have demonstrated the sustainability of using sensors for variable-rate-input applications in different crops.

Li et al. (2016) [199], for example, utilized a Crop Circle 210 crop reflectance sensor for variable rate nitrogen application (VRNA) in a corn field in Missouri. The amount of fertilizer used decreased 11% without affecting grain yield. The GWP, soil acidification, and freshwater eutrophication were reduced by 7, 10, 22, and 16%, respectively. In another study, Ehlert et at. (2004) [200] utilized a mechanical sensor for VRNA in a winter wheat field; the results showed a fertilizer reduction of 10–12% without compromising yield and grain quality. Some studies ([201,202]) have demonstrated that profits coming from the use of sensors for VRNA range from 10 €/ha to 25 €/ha, depending on the sensor and the size of the farm; more benefits come to farms bigger than 250 ha. The use of sensors for AI and machine learning applications can bring environmental and economic benefits, as illustrated by precision agriculture technologies. It will be relevant to have decision support tools such as LCA to assess the sustainability of such technologies in the future but at the same time make use of such technologies to collect the data needed to perform such assessments. In the long run, AI will most certainly lead to a transformation of entire business practices and industries toward a more sustainable path, by simultaneously fostering and facilitating environmental governance [203].

Despite the strong media interest in the digital transformation in agriculture, implementation in practice in the alpine region is not far advanced yet. Limiting factors are high investment requirements, a lack of integration into existing agricultural systems, and the lack of training of stakeholders in the sector [204,205]. The technologies available on the market allow precise intervention in agricultural processes, but usually use only single parameters in online transactional processes (OLTP), which are based on simple linear models [206]. However, agriculture is governed by naturally complex processes, which usually need to be represented using a large number of parameters with nonlinear relationships [207].

With the transition from precision agriculture, where only variability on land is considered, to smart farming, which emphasizes the use of complex structured and dynamic information and communication processes in farm management, new technologies, such as computer vision, big data, Internet of Things (IoT), cloud computing, robotics, and artificial intelligence (AI), are entering agriculture. Based on these technologies, digital transformation requires solutions according to online transactional processes (OLAP), which can make decisions based on a wide range of parameters [206,208,209]. Sensor development and information acquisition provide the essential foundation for this. Building on this, precision agriculture faces the challenge of requiring ever higher application precision. Technological developments in automation over the past few decades in agriculture have significantly increased the productivity of agricultural machinery by increasing efficiency, reliability, and precision; and reducing the cost of production and manual, strenuous field labor [173]. Robotics offers the opportunity to further increase both precision and efficiency [210].

Human–machine interaction always faces safety issues in the context of any type of autonomous navigation. The safety of humans, animals, and objects is a key requirement in the automation of agriculture and forestry. While for some industrial robots, it is sufficient to delineate the workspace, in the agricultural sector, a higher level of safety is required due to the more direct physical interaction [211].

Computer vision, i.e., imaging sensors and methods for analyzing images, is already being used in various areas of agriculture and food production [212,213]. On the one hand, this concerns applications in environment-dependent/dynamic navigation (including safety aspects), and on the other hand, information retrieval for process control and process evaluation.

Machine or deep learning algorithms are mainly used at first for classification, localization, and detection of different plants in the form of artificial neural networks [214,215]. This requires sufficiently annotated and high-quality training data, which can be immensely costly to produce and has been very limited in (free) availability in the field of precision agriculture [78,216]. Therefore, research is also being conducted on solutions using non-annotated data and semi-supervised learning [217]. In addition, hyperspectral imaging has gained importance in recent years. However, since cost-effective applications are not yet available, research continues on systems with only one measurement point for plant characterization [218]. All of those systems generate large amounts of data. In addition, data from sensors are becoming increasingly available in machines and devices in the context of stationary data (IoT), which could provide additional process/system parameters. Big data technologies, where large amounts of data with great variety are collected and analyzed, provide access to explicit information, and can therefore contribute greatly to decision-making processes through modeling and optimization. By combining big data with other external data sources, such as weather or market data, the benefits can be significantly increased. However, gaining knowledge from big data usually requires novel methods and special techniques that are often diverse and complex [219,220].

Wolfert et al. (2017) showed that information technologies in agricultural engineering are generally still at an early stage of development. The large amounts of data, however, not meeting i.i.d. requirements, pose a particular challenge [221]. It is expected that through access to real-time data and real-time forecasts, and through combination with IoT developments and especially AI, technologization will advance. While this prospect is promising, challenges such as data quality, privacy, and security issues cannot be neglected, arguing for a synergistic approach of human-centered AI to reconcile new technologies in agricultural technology with human values, ethics, and legal requirements to ensure privacy, security, and safety.

Future developments will increasingly provide data and information along the entire value chain in real time (challenge to the capacity of telecommunications-6G [222]) and with location accuracy (challenge to the precision of telemetry, [223]). In the context of sensor developments (increases in diversity, sensitivity, robustness, and precision, while decreasing prices), a basis will be created that allows agricultural processes to be recorded in a more differentiated way based on data. By using the experiential knowledge of humans via human–machine–human communication (human-in-the-loop, AI-in-the-loop), technical processes can be much more finely adapted to natural processes, which will contribute to increasing the resource efficiency of production processes (“feed the world”), and to improving the quality of products, both internally (human health) and externally (e.g., life cycle impact, animal welfare, environmental protection, and climate protection). In this context, networking all stakeholders through appropriate technology can increase the transparency of food production, improve documentation, and ultimately improve traceability—these are general goals of human-centered AI, and here we are again speaking of explainability. To achieve these benefits, AI tools that are appropriate, and above all, developed in a human-centered way (see Section 4.2), will play a crucial role in linking technologies and using domain knowledge, for natural agricultural systems, increasing their global and local diversity, and helping their sustainable management.

Therefore, data-driven AI applications can help to overcome the sectoral fragmentation of IT applications in agriculture and create links between agriculture, food processing, and consumers on the one hand, and the industry supplying agriculture on the other (e.g., predictive maintenance of machinery).

- Open Challenge G1: Identify a requirements map and technology overview. Create a toolbox of existing technologies for inexperienced farmers with easy-to-use methods and cost-effective applications to create benefits in everyday life according to the concept of human-centered design (see Section 4.2).
- Open Challenge G2: Making online available data accessible and integrate the structure and computational operations of the above toolset into AI solutions. Networking, fusion, integration, presentation, and visualization of information from different sources and locations, following the information visualization mantra “overview first, zoom and filter, then details on demand” [224] to provide a respective snapshot across the entire value chain, thereby identifying insight and opportunities for further analysis of key indicators across the entire value chain (“from seed to the consumer’s stomach”).
- Open Challenge G3: A key challenge is to gradually refine the tools of LCA, LCC, and SLCA.

### 4.4. Forester-in-the-Loop

Forest education in Austria is based on the general Austrian education system and includes various forestry professions. The theoretical and practical education, imparted through school, university, an apprenticeship, or specialized courses, ensures the competence of the forestry personnel of tomorrow and forms the foundation of Austrian forestry. After a three-year apprenticeship, secondary courses, or an exam following agricultural and forestry colleges, forest workers are optimally trained for motor-manual forest activities in reforestation, cultivation, maintenance, and harvesting. Since 2016, it has been possible to learn the profession of forestry technician, where the main focus is on the handling of equipment and machinery for timber harvesting. This solid education in combination with the theoretically and practically acquired expertise is very well suited to be integrated into forestry AI processes. In this way, the expertise and practical experience of the forester/forestry worker/operator acquired in school can contribute to the training of AI systems, and conversely, the human can be supported and relieved of their decisions and activities.

In steep terrain, especially in Austrian forests, timber harvesting will always be a challenge in terms of economic viability, safety, and environmental performance. The state-of-the-art harvesting method in steep terrain is motor-manual felling in combination with logging via cable yarders. Even though new technologies and innovations are modernizing timber harvesting in steep terrain, there will always be operations where forest workers need to enter the forest due to the difficult topological conditions. In these cases, it is the task of human-centered AI to intelligently integrate workers on the site into already autonomous or semi-autonomous timber harvesting operations. For example, potentially autonomous cable yarders must interact appropriately with workers on the site, and both parties must be considerate of and learn from each other.

All forestry machines, whether harvesters, forwarders, cable yarders, or simply chainsaws, and whether autonomous, automated, remote, or traditionally controlled by a human in the cab, have certain maintenance and repair needs. Some repairs also occur randomly and suddenly, such as chain breakage on a harvester head or replacement of a hydraulic hose. These repairs are then usually carried out by the operator on site. In future AI-supported autonomous or automatic processes, humans must also be involved in this topic with their expertise in all aspects of the repair. Real-time anomaly and fault detection (see Section 3.1) is faster, more reliable, and needs less visualization capabilities [145]. This is going to be of great benefit to the user, since this will tackle efficiently the problem of filtering out information (see Section 3.3), while at the same time detecting the faults efficiently and needing the domain expert only for the definition of normal vs. abnormal behavior and potential repair on-site.

Information about AI in forest operations can also be found on websites and in popular science journals. However, science has basically been dealing with developments of robotics in forestry since the 1980s (as in [225]). Over the years and with increasing sensor technology and computer power, a number of forest operations research groups are experimenting with digitization in forest operation. Visser and Obi (2021) [88] stated that there is often much speculation on future benefits, and there is almost a complete absence of information on actual productivity improvements of any of the prototypes developed. For forestry practice, it can be stated that there is a lot of R&D in the field, but the developments have not actually found their way into the real forest yet. The next steps to successfully establish new technologies and AI in timber harvesting are to use existing technologies, which are already well equipped with sensors and computing power, to link them with AI and to integrate the forest worker as an integral control, steering, and assistance organ.

- Open Challenge G1: Due to the small-scale forestry in Austria, many forest owners work in their own forests. Therefore, most of the work is done manually with a chainsaw. The future task of the forester-in-the-loop approach is to develop digital and smart tools in this area as well. For example, individual tree information could be shown to the forest owners via heads-up displays in their helmets or VR glasses. Furthermore, it would also be conceivable to provide assistance with regard to value-optimized bucking in order to increase efficiency and revenue. Conversely, the AI can always learn from the forestry worker’s expertise and concrete actions during the work process.
- Open Challenge G2: In principle, a good first step into autonomous practice would be to decouple the acquisition of the environment data of a forest machine and its AI-controlled features in terms of time. For example, digital twins of the forest can be created with 3D scanners (often takes place as part of forest inventory anyway); and autonomous, automated, or even augmented processes can be integrated into the forest machine on the basis of these. This saves time-consuming on-the-fly environment mapping and navigation. Robots (e.g., [226]) offer a good opportunity to test such a process. During the entire process, the forester should be specifically involved and make decisions.
- Open Challenge G3: Based on Open Challenge G2, a direct navigation system for forestry machines should be developed, enabling on-the-fly navigation. This concerns not only the pure driving with the machine but also the autonomous control of attachments and aggregates (e.g., crane and harvester head). This not only applies to simply driving the machine, but also to the autonomous control of attachments and aggregates (crane, harvester head, etc.). In this further development step, it is also necessary to integrate further sensors and to process their data intelligently (GNSS sensor, 3D laser scanners, 2D laser scanners, stereo cameras, hydraulic sensors). Furthermore, the methods and approaches should also be transferred from the small robot to a real forest machine. The expertise of the previous operators should be incorporated in order to avoid environmental impacts and to work in a value-optimized manner. This step towards a large forestry will be done in close cooperation with machine manufacturers. Furthermore, large-scale practical studies will be carried out in the forest in order to test and continuously develop the systems.

## 5. Conclusions

Advanced technologies—from sensors to sophisticated augmented reality visualization methods—are nowadays already so inexpensive that they are very widely used. As a result, they are already being used as decision support tools in a wide range of application situations. The hardware is available; what needs to be worked on now is bringing intelligence into the hardware. This is where human-centered AI comes in—namely, not only providing the domain experts with contextual and relevant information, but also involving them directly in the decision-making process. A human expert often has a lot of experiential knowledge, and it can be useful to combine this natural intelligence with artificial intelligence.

In our paper, we first justified why agriculture and forestry are among the most important application areas for all humankind. Then, we facilitated a common understanding by providing definitions of artificial intelligence and human-centered AI, and introduced the three main approaches to machine learning (supervised, unsupervised, reinforcement). This should help the non-expert reader to get started with this important and forward-looking topic. We then presented the current state-of-the-art in autonomous, automated, assisted, and augmented AI systems. The special feature here is that we gave one example from agricultural technology and one example from forestry technology, thereby helping to understand the connection between these two areas.

In this paper, we described three pioneer research areas that we identified as the most important and promising research areas for the next 7 years based on our experience: (1) intelligent sensor information fusion, (2) robotics and embodied intelligence, and (3) augmentation, explanation, and verification. Finally, we summarized again the core ideas of human-centered AI and gave two examples of farmer-in-the-loop and forester-in-the-Loop. We are convinced that the next 7 years will be internationally dominated by these topics, and that with their help, practical progress can be made in the entire process chains of future agriculture and forestry.

## Data Availability

Not applicable.
